# Coagulopathy in viral haemorrhagic fevers and beyond: molecular mechanisms and targeted interventions

**DOI:** 10.3389/fmolb.2026.1804180

**Published:** 2026-06-26

**Authors:** Nabh Chandra, Dhruv Rao, Yuvaraj Sivamani, Nimmy Srivastava, Dibyajit Lahiri, Moupriya Nag, Sunitha C. Mesta, Sumitha Elayaperumal

**Affiliations:** 1 Department of Biotechnology and Bioinformatics, School of Life Sciences, JSS Academy of Higher Education and Research, Mysore, India; 2 Crescent School of Pharmacy, B.S.Abdur Rahman Crescent Institute of Science and Technology, Chennai, Tamilnadu, India; 3 Amity Institute of Biotechnology, Amity University, Ranchi, Jharkhand, India; 4 Department of Basic Science and Humanities, Institute of Engineering and Management, Kolkata, Salt Lake Sector, University of Engineering and Management, Kolkata, India; 5 Department of Microbiology, School of Life Sciences, JSS Academy of Higher Education and Research, Mysore, India

**Keywords:** coagulopathy, immune response, microvascular thrombosis, thromboinflammation, tissue factor (TF)

## Abstract

Coagulopathy refers to any medical condition which affects the ability of the blood to clot. It can be caused due to genetic conditions like haemophilia, von Willebrand disease, or it can be caused through liver disease or deficiency of Vitamin K. It also involves a broad range of diseases affecting hemostasis as an unbalanced and even bidirectional relationship between thrombosis and bleeding. Coagulopathy can also be caused by thromboinflammation, as seen in VHFs like Ebola, Dengue, Marburg, Crimean-Congo Hemorrhagic Fever, Yellow Fever, and Hantavirus infection. The immune response and coagulation system are intricately linked in such cases. Infections from VHFs cause endothelial cell dysfunction through the immune response, monocytes/macrophages activation, and increased expression of tissue factor (TF), which in turn causes excessive thrombin production and fibrin formation. These conditions result in microvascular thrombosis, organ dysfunction, consumption of platelets and coagulation factors, causing a balanced but fragile state of hemostasis that could tip over towards either thrombosis or bleeding. New therapies have been developed that interfere with these processes, such as interference with the TF pathway (for instance, rNAPc2) and regulation of fibrinolysis (tranexamic acid). The recognition of the double-edged sword of coagulopathy is critical for the development of treatment strategies targeting coagulation disorders. This literature review discusses the molecular basis of immunothrombosis and endothelial dysfunction in VHFs.

## 1 Introduction

Viruses that cause viral hemorrhagic fever (VHF) include Lassa virus (Lassa fever), Marburg virus (Marburg fever), and Ebola virus (Ebola fever) (Table 1). VHFs are highly systemic diseases involving endothelial cell dysfunction with vascular leak, as well as an immune response and a hypercoagulable state. VHFs were traditionally considered due to their hemorrhagic complications, but today, scientists prefer a broader definition based on the phenomenon of thromboinflammation, which is the interplay between immunity and coagulation factors. VHFs are caused by viruses from various genera and families, such as Ebola virus, which belongs to the family Filoviridae. It attacks monocytes, macrophages, and dendritic cells, activating innate immunity pathways strongly. Pro-inflammatory cytokines like IL-1β, IL-6, and TNF-α are released together with chemokines IL-8. These factors promote endothelial cell activation and not endothelial cell injury (Meltzer, 2012; Schnittler and Feldmann, 2003).

**TABLE 1:** Viral haemorrhagic fever–associated viruses and their coagulopathic profiles.

Virus	Viral family	Primary target cells	Key hemostatic abnormalities	Main hemostatic phenotype	Clinical manifestations	Case fatality rate (cfr)
Ebola virus	Filoviridae	Monocytes, macrophages, endothelial cells	DIC-like coagulopathy, tissue factor overexpression, dysregulated fibrinolysis (Meltzer, 2012; Schnittler and Feldmann, 2003)	Consumptive + thromboinflammatory	Mucosal bleeding, internal hemorrhage, multiorgan failure	25%–90% (varies by outbreak and care settings) (Palta et al., 2014)
Marburg virus	Filoviridae	Macrophages, endothelial cells	Severe consumptive coagulopathy, thrombocytopenia (Petäjä, 2011)	Consumptive (severe DIC phenotype)	Extensive hemorrhage, shock	∼24–88% (outbreak-dependent variability) (Palta et al., 2014; Petäjä, 2011)
Dengue virus	Flaviviridae	Monocytes, dendritic cells, megakaryocytes	Thrombocytopenia, platelet dysfunction, capillary leak (Lamperti et al., 2025; Schmid-Schönbein, 2006)	Platelet-dominant + permeability-driven	Epistaxis, gum bleeding, GI bleeding, plasma leakage	<1% with appropriate management (Schmid-Schönbein, 2006)
Lassa virus	Arenaviridae	Endothelial cells, macrophages	Endothelial dysfunction, impaired platelet aggregation (Zapata et al., 2014)	Permeability-driven	Mucosal and internal bleeding, vascular leak	∼15–20% (higher in hospitalized patients) (Zapata et al., 2014; Marcos-Jubi et al., 2023)
Crimean-Congo HF virus	Nairoviridae	Endothelial cells, hepatocytes	Prolonged PT/aPTT, fibrinolysis, platelet consumption (Sharma et al., 2026)	Mixed: consumptive + hyperfibrinolytic	Severe hemorrhage, ecchymosis, organ dysfunction	10%–40% (region and care dependent) (Sharma et al., 2026; Iba et al., 2020)

One of the important mechanisms of VHF pathogenesis involves TF induction on monocytes and endothelial cells, leading to activation of the extrinsic cascade of coagulation, resulting in thrombin production and fibrin deposition. In turn, thrombin activates inflammation by engaging protease-activated receptors (PARs), forming a positive feedback loop between inflammation and coagulation systems. Infection-induced activation of such processes may cause thrombosis of small vessels and organs’ dysfunction, which characterizes severe forms of disease. It should be emphasized that infection-related coagulopathy in VHFs is far from being a straightforward issue associated with hemorrhage. On the contrary, this process should be understood as the re-establishment of hemostasis in conditions of increased tendency both for excessive clotting and bleeding. The early phases are often characterized by procoagulant features with microthrombi formation, whereas the late ones–by consumption coagulopathy and hemorrhages, typical for DIC (Schnittler and Feldmann, 2003).

This is a critical step towards developing therapies. The approach targeting major pathways like transcription factor activity, thrombin production, vascular dysfunction, and fibrin degradation has shown great promise. The aim of this study is to investigate the molecular basis of thromboinflammation and coagulopathy in VHFs.

## 2 Overview of coagulopathy in infection

### 2.1 Physiological coagulation pathways

Coagulation is a vital process which helps to understand the blood coagulation system which plays an important role for anaesthetic practice. Classification of the coagulation is usually done by extrinsic pathway and intrinsic pathway. A balance in the coagulation system is maintained by a procoagulant pathway which has a main role in formation of clot and the mechanism responsible for the inhibition which extends beyond the injury site. Imbalance of this system occurs during critical illness which can cause thrombosis or bleeding (Palta et al., 2014).

Physiological coagulation is a multiple step complicated pathway that ensures effective hemostasis while preventing excessive blood clotting. Hemostasis is a process that arrests bleeding. This process occurs due to the presence of balance and interaction between coagulation, platelets, fibrinolytic systems and vessel walls. Hemostasis can be classified in two types: primary and secondary hemostasis (Palta et al., 2014; Petäjä, 2011).

#### 2.1.1 Primary hemostasis

Primary hemostasis is a process in which interaction of vessel walls, platelets and adhesive proteins occurs, this interaction helps in the formation of the “platelet plug” (Lamperti et al., 2025). This step is also known as platelet plug formation. The presence of negative charged heparin glycosaminoglycans which contributes to its antithrombotic properties in the vascular wall of endothelial cell lining. In normal conditions platelets do not adhere to intact vascular endothelium, but when there is vascular injury, they adhere to the collagen and von Willebrand Factor (vWF) in sub-endothelial tissue and morphologically changed is observed in them which form irregular surface with pseudopods which increases their surface area (Lamperti et al., 2025). Platelet adhesion is promoted by vWF which serves as a bridge between platelet surface receptors Gplb and endothelial collagen. Once the adhesion of the platelets is done, degradation from both alpha and dense granules occurs which releases various factors. Phospholipids which are exposed by platelet activation bind to the calcium, providing a surface for the assembly of different coagulation factors. These activated proteins are responsible for the production of Thromboxane A2 (TxA2), responsible for providing the stimulation that causes aggregation of the platelets. TxA2 and ADP together forms a platelet plug which is responsible for the formation of the platelet plug. This plug helps in sealing the site of injury for a temporary period, preventing the loss of excess blood (Palta et al., 2014; Lamperti et al., 2025).

#### 2.1.2 Secondary hemostasis

The major component for the coagulation system is the coagulation protein and they lead to complex reactions. This results in conversion of fibrinogen strands which are soluble to insoluble. Liver helps in the production of procoagulants and anticoagulants, except for III, IV, and VIII. Vitamin-K post translational-modifications of these proteins is done, enabling them to bind to calcium and other cations which are divalent in nature and participate in the formation of clotting cascade (Lamperti et al., 2025). Factor I acts as a precursor to formation of fibrin and ultimately determines the clot’s strength. Tissue Factor, also known as Factor III, is a membrane-bound procoagulant glycoprotein found in fibroblasts and subendothelial tissue. It plays an important role in the coagulation *in vivo* and it is not exposed to the blood until the vessel wall is damaged. Activated X factor, calcium, tissue phospholipids, platelet phospholipids and cofactor, factor V helps in the formation of prothrombinase complex, this complex is responsible for conversion of prothrombin to thrombin. Thrombin is activated with the help of fibrin stabilizing factor, factor XIII, also responsible for breaking down circulating fibrinogen into insoluble fibrin factor. Factor XIII covalently crosslinks fibrin polymers, responsible for making platelet plug (Palta et al., 2014).

#### 2.1.3 Coagulopathy in infection

**FIGURE 1:**
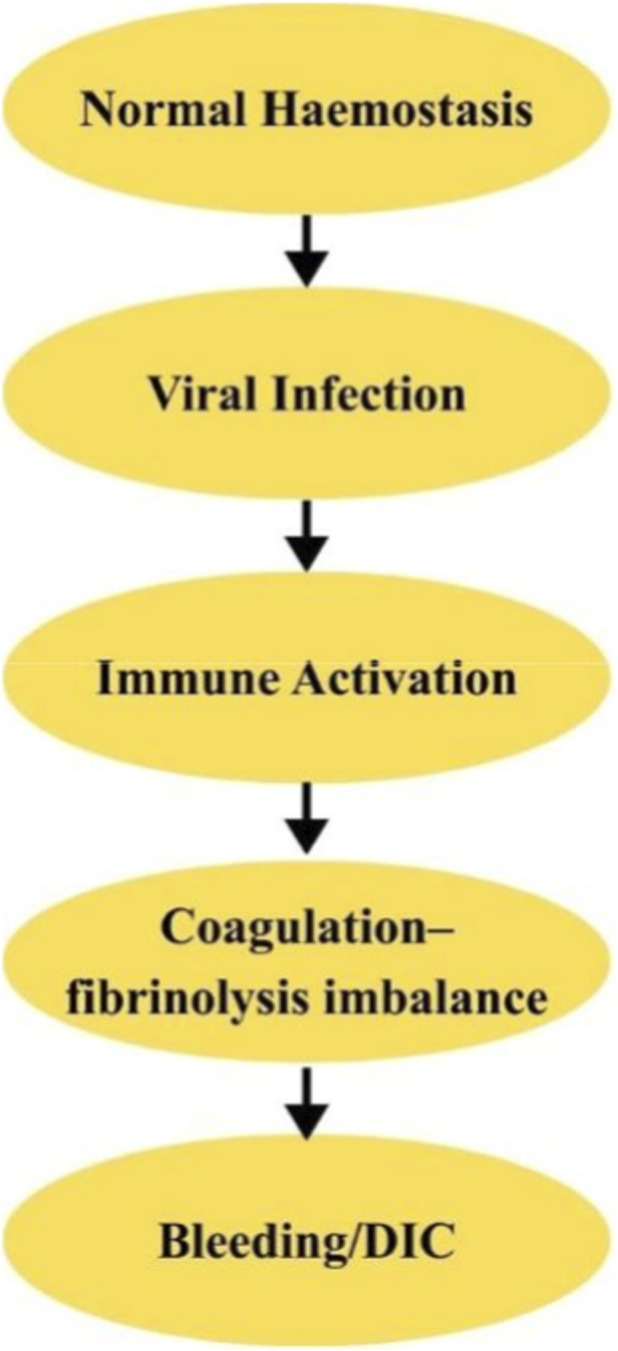
Overview of hemostatic balance and disruption during viral infection.

To maintain vascular integrity in normal physiological conditions, the hemostatic system operates as a tightly regulated network that balances procoagulant, anticoagulant, and fibrinolytic pathways. This equilibrium is maintained through coordinated interactions between platelets, coagulation factors, endothelial cells, and endogenous anticoagulant mechanisms such as antithrombin and the protein C system. However, during viral infections, this balance is intensely disrupted by inflammatory mediators, endothelial activation, and immune cell involvement. This can lead to a shift toward a prothrombotic and subsequently consumptive state. The transition from physiological hemostasis to infection-driven coagulopathy, characterised by thrombin generation, fibrin deposition, and microvascular thrombosis, is summarised in Figure 1.

### 2.2 Inflammation and coagulation

#### 2.2.1 What is inflammation?

Inflammation is a fundamental biological reaction that acts as the primary mechanism for tissue repair upon sustaining an injury (Schmid-Schönbein, 2006). It involves complex cell and microvascular processes that plays role in the removal of damaged tissue and in the regeneration of the new tissue (Schmid-Schönbein, 2006). Inflammation is usually linked in the context of infection and immunity, but new research shows its role in a wide range of diseases (Petäjä, 2011).

#### 2.2.2 Inflammatory cascade

The inflammatory cascade starts with the dilation of the blood vessels (arteries and veins), followed by increased permeability of those vessels, which increases the blood flow. This frequently leads to stasis and thrombosis, followed by leukocyte infiltration into the tissue and plasma escape. Tissue breakdown occurs as a result of proteolytic activity and the generation of oxygen-free radicals, which causes necrosis and apoptosis. Phagocytic cells then clear debris, and new humoral mediators are produced to promote cell development, resulting in the regeneration of new functional and connective tissue, a process known as resolution (Schmid-Schönbein, 2006).

#### 2.2.3 Microvascular changes during inflammation

One of the earliest signs of an inflammatory reaction is the increased permeability of the endothelium. His involves transmembrane signalling and cytoplasmic reactions in endothelial cells (Schmid-Schönbein, 2006). Adhesion molecules like selectins and integrins are expressed which facilitate the attachment of circulating cells to the endothelium (Schmid-Schönbein, 2006). Selectins govern the initial attachment, while integrins help in the firm adhesion. The neutrophils circulating the blood accumulate in the postcapillary venules, making contact with the membrane of the endothelium. This leads to firm adhesion, and eventual movement into the tissue. Cytoplasmic projection, called pseudopods, are a sign of cell activation. Pseudopods are important for cell spreading, phagocytosis and migration. In the capillaries after the reduction of blood pressure, the endothelial cells also form pseudopods. The formation of Oxygen Free Radical is an important event in cell activation. It leads to the formation of free radicals: superoxide, hydrogen peroxide and hydroxyl radicals. These are often found with neutrophils and can be detected *in vivo*. During inflammation, the mast cells release cellular contents from granules and lysosomes, in a process known as degranulation. P-selectin and vWF are released by the endothelial cells from Weibel-Palade bodies. Inflammation is often followed by apoptotic and necrotic cell death. The extent depends on the inflammatory stimulus. Significant cell death occurs during reperfusion in acute ischemia (Petäjä, 2011; Schmid-Schönbein, 2006).

#### 2.2.4 Inflammation vs. coagulation

Inflammation and coagulation are both critical physiological processes, but they serve different functions and involve distinct key mechanisms. Inflammation is primarily a tissue repair mechanism. It involves a cascade of microvascular and cellular reactions to generate new tissue and remove the damaged ones. The main goal of this whole process is to heal injury and protect the body from more harm (Petäjä, 2011).

Coagulation is the process of clotting of blood. Its main purpose is to stop the bleeding (hemostasis) and to maintain the integrity of the vascular system (Zapata et al., 2014). The coagulation factors act as mediators if the inflammation pathway. The adhesion of platelets to the endothelium during coagulation helps the adhesion of other cells to the endothelium and other cells during inflammation. This shows how coagulation and inflammation, both processes are inter-linked. The events in the coagulation pathway can contribute to or influence the inflammatory cascade. Another example is the degranulation of Weiber-Palade bodies to release vWF during inflammation, which initiates the coagulation and platelet adhesion (Palta et al., 2014).

### 2.3 Infection-induced coagulopathy and classical DIC

#### 2.3.1 Infection-induced coagulopathy

Infection-induced coagulopathy refers to an overactive coagulative system triggered by a viral infection, which leads to serious cardiovascular and thrombotic complications. Immunothrombosis refers to the relation between the immune response of the host to the viral infection and coagulation (Marcos-Jubi et al., 2023). Cytokine storm is a hyper-inflammatory response, which destabilises the vascular system and is mainly involved in the thrombotic events. The virus also activates the innate immune system of the host through Toll-like receptors. This induces the release of proinflammatory cytokines. Upon infection, viruses can damage the endothelium and can cause its surface into a pro-coagulant surface. Viruses directly infect the endothelial cells causing cell death or inflammation. This leads to the upregulation of the production of adhesion molecules and the release of vWF, promoting platelet and coagulant recruitment. Subsequent inflammation induces Tissue Factor (TF) expression on monocytes and endothelial cells. This initiates the coagulation cascade pathway (Sharma et al., 2026). During infections, platelets can engulf virions and express receptors, like TLR-7 that recognize viral RNA. For example, in influenza, platelets help in mediating the host response. In dengue, platelets release cytokines like IL 1 Beta, which increases the permeability of the endothelium. NETosis is a unique form of programmed neutrophil death that releases neutrophil extracellular traps (NETs) during the viral infections. Nets are antimicrobial proteins containing DNA-based scaffolds such as neutrophil elastase and myeloperoxidase. NETs trigger thrombo-inflammation, which can obstruct the blood vessels and promote vascular thrombosis (Iba et al., 2020). NETs act as a bridge between innate immunity and coagulation, driving infection-induced coagulopathy and thrombosis. These trap pathogens but also activate platelets and coagulation factors while inhibiting fibrinolysis. Excessive NET formation causes microvascular thrombosis, tissue damage, and Disseminated Intravascular Coagulation (DIC) in sepsis.

#### 2.3.2 Classical DIC

DIC, Disseminated Intravascular Coagulation, refers to the systemic activation of coagulation pathways, leading to widespread fibrin deposition and consumption of clotting factors and platelets. DIC is an intermediary mechanism of several underlying diseases. The clinical manifestation of DIC depends on the balance between the intravascular clotting process and secondary fibrinolysis and it ranges from diffuse thromboses to hemorrhage, or both (Loftus, 2016). DIC manifests as diffuse thromboses if the intravascular clotting is dominant and secondary fibrinogenolysis is minimal, as seen in malignancy. Conversely, if secondary fibrinolysis is dominant and pro-coagulant activity is minimal, DIC will manifest as hemorrhage, which is seen more commonly. The symptoms of acute DIC include shock, associated end-organ hypoxia, and ischemic changes. Patients with acute or sub-acute DIC often show signs of multiple hemostatic compartment defects. This often leads to melena, hematemesis, epistaxis, hematuria, petechiae, and purpura (Bick, 1978).

## 3 Pathophysiology of viral haemorrhagic fevers

VHFs can be caused by various RNA viruses, including members of arenavirus bunyavirus and filovirus families. Viral Hemorrhagic Fever is a clinical condition that contains hallmarks like restlessness, fever, vascular permeability, depletion of plasma levels, problem in coagulation of blood and different degrees of bleeding (Basler, 2017). Common symptoms of VHFs can include chills, fatigue, nausea and vomiting (Schnittler and Feldmann, 2003). Coagulation abnormalities are also observed in the patients which generally includes prolonged prothrombin time and partial thromboplastin time. It has been seen that damage to the liver can affect the production of clotting factors, contributing to hemorrhage. Ebola virus, a member of the filovirus, is the most extensively studied VHF agent (Basler, 2017; Chang, 2026). The mechanism of the virus is discussed below.

### 3.1 Virus-host interaction

VHFs invades the host through mucosal surfaces or compromised skin integrity as observed in Ebola virus, and can also enter with the help of infected rodent excreta (Lassa virus) or insect bites (yellow fever virus) (Basler, 2017). Macrophages and dendritic immune cells are targeted by the EBOV in the initial stage of infection; in later stages, almost all cells are affected except for lymphocytes. The virus enters the target cells through various uptake processes such as, macropinocytosis and raft mediated endocytosis. For the uptake of EBOV, the phosphatidylinositol-3 kinase-Akt pathway and cytoskeletal protein dynamics are essential. EBOV attaches itself to the cell surface receptors with the help of viral glycoprotein and it is internalised and moved via early and late endosomes. Host factors are important for EBOV’s membrane fusion and escape from vesicular compartments; these host factors are the homotypic fusion and vacuole protein sorting (HOPS) multisubunit tethering complex and Niemann-Pick C1 (NPC1) protein (Chang, 2026). It has been observed, the patients whose cells are deficient in Hops and NPC1 protein are resistant to Ebola infection (Chang, 2026).

Glycoprotein 2, which is formed during the proteolytic cleavage of GP1 by endosomal proteases cathepsin B and L mediates the viral and cellular membrane fusion. After the fusion, the cytoplasm, where viral genome transcription and replication take place, receives the viral nucleocapsid. Infection of Ebola virus leads to dysfunction in innate system immune cells. Interferon type-1 (IFN) production and response is inhibited by the virus, also perturbs cytokine networks and impairs the function of dendritic cells (DC) and natural killer cells (NK) (Raj et al., 2025). IFN and DC maturation inhibition is observed in Lassa virus also (Raj et al., 2025). The nucleoprotein of Lassa virus blocks IFN induction, partly with the help of its 3′-5′ exonuclease activity, it also inhibits RIG1 and MDA5 (Falasca et al., 2015).

Other than these viruses, yellow fever virus also inhibits IFN responses through proteins like NS4B (Basler, 2017).

### 3.2 Immune activation and cytokine storm

Ebola virus (EBOV) infection triggers a significant and often detrimental immune activation, characterized prominently by a “cytokine storm.” During initial immune response the shed from glycoprotein (GP) is released from the surface glycoprotein and then it binds to and activates the uninfected dendritic cells and immune cells with the help of TLR4 engagement (Chang, 2026; Falasca et al., 2015). This leads to secretion cytokines, which can be either pro or anti-inflammatory in nature (Falasca et al., 2015). The mechanism of the activation of the immune system in uninfected cells contributes to systemic inflation and the cytokine storm (Falasca et al., 2015). Studies conducted *in vitro* demonstrated that EBOV infection can cause PBMC or monocytes/macrophages to produce large amounts of cytokines and chemokines (Falasca et al., 2015).

This phenomenon is a checkpoint of non-specific immune action to the Ebola virus. Numerous pro-inflammatory cytokines are secreted during the innate immune response, leading to cytokine storm. This damages the immune cells by generating a plethora of conflicting signals.

While fatal infections are linked to a dysregulated inflammatory immune response, patients who recovered from Ebola infection displayed an fast and transient increase in the cytokine level present in the serum. This implies the activation of the innate immune response (Falasca et al., 2015). In recent studies it has been seen that fatal infection acquires exceptionally high levels of chemokines (MIP-1a, MIP-1β, MCP-1, MIF, IP-10 GRO-a, and eotaxin) and cytokines like IL-1β, IL-1RA, IL-6, IL-8, IL-15, and IL-16, which are pro-inflammtory in nature, (Table 3). Their levels started to rise soon after the disease started and continued to rise until the death of patients (Falasca et al., 2015). Survival appears to be linked to a delayed increase in viral RNA level in the serum that aligns with prolonged cytokine and chemokine response (Falasca et al., 2015). IL-10 plays an important role in the regulation of inflammation, also known as immunomodulatory cytokine. Survivors had slightly higher levels of IL-10, most likely as a process to regulate the inflammatory response. This increase was transient, as would be expected after cytokine levels returned to normal. However, in fatal cases, IL-10 was six to ten times higher and stayed elevated until death (Basler, 2017; Rivera et al., 2016).

#### 3.2.1 Consequences of dysregulated immune activation

Inflammatory mediators and chemokines produced by EBOV-infected DC and macrophages can attract more DC and macrophages to infection sites, increasing the number of cells that the viruses can invade and infect. This intensifies the dysregulated response of the host (Falasca et al., 2015). Soluble mediators such MCSF, MIP-1a, IP-10, and sICAM have been linked to hemorrhagic manifestation. According to theory, these cytokines may attract leukocytes to inflammatory regions. The synthesis of ICAM (or other adhesion molecules) promotes leukocyte attachment, diapedesis and rolling (Falasca et al., 2015). An active, leukocyte-enriched, procoagulant endothelium would remain as a result, leading to dysregulated hemostasis, which could show up clinically as bleeding (Falasca et al., 2015). Overall, it appears that the production of these mediators caused by viruses leads to an immune imbalance, which aids in the pathophysiology and advancement of the disease (Basler, 2017; Rivera et al., 2016).

### 3.3 Capillary leak and endothelial injury

Capillary leak is also known as vascular leakage or permeability. It is a critical feature of VHFs and is an essential component of their pathogenesis. This phenomenon has a significant contribution to the serious clinical manifestations and high fatality rates seen in these diseases. It is characterized by increased permeability of the vascular system. The fluid and the plasma get released from the blood vessels to the surrounding tissues. It leads to a decrease in plasma volume, which results in low blood pressure (hypotension) and can eventually cause circulatory collapse or even death over the duration of the infection (Basler, 2017). Symptoms of VHFs, like Ebola virus disease, are characterized by restlessness, fever, loss of permeability of blood vessels, reduction in plasma volume, abnormal clotting of blood, and internal bleeding (Basler, 2017). The endothelial cells form the inner lining of the blood vessels and play an important role in maintaining the vascular integrity. Injury to the endothelium leads to capillary leak. The mechanisms that contribute to capillary leaks and endothelial injuries include inflammatory responses, production of cytokines, damage to the endothelium, and coagulation abnormalities. Cytokine storms, which are an inflammatory response induced during a VHF, promote leakage of the blood vessels and coagulopathy (Basler, 2017). Cytokines such as TNF (Tumor Necrosis Factor) are produced during Ebola infection, and it promotes endothelial leakage. The Ebola virus targets monocytes and macrophages, which then produce damaging cytokines that contribute to vascular leakage. The excessive production of cytokines can also upregulate the coagulation process that leads to DIC (disseminated intravascular coagulation) (Basler, 2017). This indicates direct damage to the endothelial cells. Tissue Factor (TF) produced during an inflammatory response in immune cells like the monocytes and macrophages activates coagulation cascades and causes DIC. This damage to the coagulation system and the increased levels of fibrin degradation products result in the breakdown of the vascular system. Infection of macrophages and dendritic cells promotes productive viral multiplication and systemic spread. This systemic dissemination and injury to multiple tissues and organs causes the excess cytokine responses, vascular leakage, and coagulopathy which are seen in VHFs (Basler, 2017).

### 3.4 Organ-specific manifestations

#### 3.4.1 Liver damage

Patients with Ebola Virus Disease (EVD) often show signs of damage in liver, showing an elevated serum alanine and aspartate aminotransferase (ALT, AST) level (Basler, 2017). Patients with Yellow Fever Virus (YFV) also showed higher AST and ALT levels. Liver damage can lead to decreased production of fibrinogen and clotting factors, contributing to coagulation defects. Severe jaundice is a distinct feature of YFV, not usually seen in other VHFs. Lassa virus also infects the liver, and escalated levels of ALT and AST are usually seen in affected individuals (Basler, 2017).

#### 3.4.2 Lymphoid organ damage and immune dysregulation

Lymphopenia is a feature unique to severe infection of Ebola Virus (Basler, 2017). It refers to a reduction in lymphocytes. This is accompanied by decreased levels of lymphoid and death in the spleen, thymus, and lymph nodes in lethal infections in humans and non-human primates (NHPs) that were infected experimentally (Basler, 2017). The reduced levels of lymphocytes is most pronounced in T-cells and Natural Killer (NK) cells (Basler, 2017). The dendritic cells (DCs) which are infected does not maturation properly, which affects the acquired immunity. Ebola impairs DC maturation, function, which is important for initiating adaptive immune responses. The virus that causes Lassa fever similarly stops the response and the maturation of IFN and DC respectively, failing to activate DCs or to create a strong T-cell response. Macrophages and monocytes are commonly targeted during infection and dysregulation of the immune system (Basler, 2017). Infected monocytes and macrophages produce damaging inflammatory cytokines, contributing to disease manifestation (Basler, 2017).

#### 3.4.3 Circulatory system and coagulation abnormalities

Unchecked replication of the virus and the inflammatory responses promote leakage in the vascular system and abnormal coagulation. Patients with EVD show coagulation defects, as seen by delayed prothrombin time (PT), partial thromboplastin time (PTT), and hemorrhage (Basler, 2017). Elevated D-dimers are detected, indicating fibrin degradation. Thrombocytopenia (low platelet count) and consumption of clotting factors also indicate impaired coagulation. Decreased plasma volume can lead to hypotension, that can lead to circulatory and can be life-threatening. This is seen in YFV, where cytokine dysregulation leads to lymphopenia, endothelial damage, DIC, and circulatory shock (Basler, 2017).

#### 3.4.4 Kidney involvement

Pathology specimens from autopsy show tubular necrosis in the kidneys. Hemorrhagic Fever with Renal Syndrome (HFRS) is a type of VHF caused by bunyaviruses, which mainly affects the kidneys (Basler, 2017; Kim et al., 2025).

#### 3.4.5 Neurological manifestations

Lassa virus is known to significantly affect the brain, causing hearing loss, body tremor, restlessness, inflammation, and seizures (Basler, 2017).

## 4 Molecular mechanisms of coagulopathy in VHFs

### 4.1 Endothelial dysfunction

Endothelial dysfunction means impaired function of the cells of the endothelium which are found in the inner surface of the blood vessel (Fosse et al., 2021). In many viral diseases this dysfunction is a central feature and can lead to severe health complications. In endothelial dysfunction bioavailability of nitric oxide is severely affected, which is crucial for regulating blood flow and other endothelial functions. A non-invasive test is used to measure NO-mediated response and define endothelial dysfunction, known as flow-mediated dilation (FMD) of the Brachial Artery (Fosse et al., 2021). An endothelial phenotype that supports the protective host response is stimulated by tissue damage or infection, and endothelial cells regulate vascular activities (Fosse et al., 2021). However, endothelial dysfunction may result from excessive or persistent activation. These cells mainly maintain blood fluidity, control plasma protein extravasation and also manage leukocyte trafficking. Pathology occurs when these functions are disrupted. Signs of dysregulated vascular function are frequently present in viral diseases. Dysregulated blood flow, unchecked vascular permeability and inflammation, microvascular thrombosis, and bleeding are common manifestations (Fosse et al., 2021) (Table 2).

**TABLE 2:** Endothelial dysfunction and platelet abnormalities in VHFs.

Pathological process	Molecular mediator	Effect on platelets/endothelium	Clinical consequence
Endothelial apoptosis	Caspases, ROS	Loss of vascular integrity	Plasma leakage
Glycocalyx degradation	Heparanase	Reduced anticoagulant surface	Bleeding tendency
Platelet apoptosis	Mitochondrial pathways	Reduced platelet count	Thrombocytopenia
Platelet consumption	Immune complexes	Accelerated clearance	Haemorrhage
Impaired platelet production	Megakaryocyte infection	Reduced thrombopoiesis	Prolonged bleeding

#### 4.1.1 Direct viral damage

The pathophysiology of many viral illnesses includes direct viral damage to endothelial cells, which can result in vascular dysfunction and serious consequences. Numerous methods can result in this direct influence. Patients who had severe COVID-19 exhibited viral inclusion bodies in the endothelium, endothelial cell death, and endotheliitis, which indicates direct viral damage to the endothelial cells (Fosse et al., 2021). Multiple organs are affected by these morphological changes, including lungs and gastrointestinal tract, giving us insight of widespread endothelial activation and damage. Viral infections can lead to extensive vasculitis, severe endothelial injury with segmented destruction, mural necrosis, fragmented nuclei, and hemorrhages adjacent to damaged endothelium (Fosse et al., 2021).

Viral damage involves infecting and replicating within endothelial cells. These viral proteins manipulate host cell pathways or they directly degrade the component of the endothelial layer, contributing significantly in pathology of viral diseases (Fosse et al., 2021).

#### 4.1.2 Loss of barrier integrity

A protective lining is present in the blood vessels primarily composed of endothelial cells; breakdown of this lining is known as loss of vascular barrier integrity. This disruption causes fluid, solutes and macromolecules to move from bloodstream to surrounding tissues, causing conditions like edema and hemorrhage, which are commonly seen in viral diseases.

Crosstalk between the glycocalyx and intercellular junctions, two essential endothelium components, is necessary for the preservation of microvascular integrity. The glycocalyx covers the outer layer of every vascular cell of endothelium. Acidic oligosaccharides and terminal sialic acids give this thick, hydrated layer of glycoproteins a net negative charge. This thick layer plays a crucial role for maintaining proper barrier function, disruption of this layer can lead to vascular leakage. A key element of adherens junctions, the highly endothelial cell-specific vascular endothelial (VE)-cadherin plays a critical function in controlling the permeability to plasma proteins across intact endothelium (Fosse et al., 2021).

Some viruses target and directly manipulate intracellular junctions, which can lead to severe disruption of vascular barrier. For reference, hantavirus is responsible for dysregulation of vascular permeability by directly affecting the intercellular junction (Fosse et al., 2021).

Viral proteins that are secreted can directly damage endothelial glycocalyx. An example of dengue can be considered as it is extensively studied for this case, it has a non-structural protein (NS1) responsible for inducing the removal of the pulmonary endothelial glycocalyx by increase in endothelial cathepsin L, sialidases, and heparanases, resulting in vascular permeability (Fosse et al., 2021).

Viral infection or tissue damage can often trigger the type 1 and type 2 stimulation of the cells of endothelium which increases permeability of the vascular system (Fosse et al., 2021). Normal vascular function is disrupted by excessive endothelial NF-kB activation and Type 1 interferon signaling and contributes to vascular dysfunction (Fosse et al., 2021).

### 4.2 Mechanisms of thrombocytopenia

Thrombocytopenia is common during many viral infections, and it is characterized by a low platelet count of less than 150 × 10^9/L (Gresele et al., 2017). Platelets play an active role in the immune response against viral infections (Raj et al., 2025). The mechanism of thrombocytopenia involves several pathways. Excessive platelet consumption can occur in forming blood clots due to inflammation. Platelets can also be sequestrated from the circulation through phagocytosis and hypersplenism (Page and Liles, 2013). The immune response of the host to a viral infection can lead to inflammation, activating platelets. Platelets can also bind to neutrophils and trigger phagocytosis. Viruses can activate the coagulation system, leading to the generation of thrombin and activation of platelets (Azeredo et al., 2015). Immunoglobulins that are produced by the B-lymphocytes can cross-react with the platelet surface integrins, which can cause immune thrombocytopenia. Platelets can directly be activated by circulating viral particles, which causes an increase in adhesion molecules, degranulation, and formation of thrombi. Viruses may also indirectly infect platelets, leading to their destruction (Raadsen et al., 2021). Another mechanism is platelet-leukocyte aggregation, followed by phagocytosis by the macrophages (Zapata et al., 2014). Activated platelets recruit leukocytes to the inflamed endothelium (Zapata et al., 2014). This is mediated by proteins like platelet GPIbα and the leukocyte MAC-1 (Zapata et al., 2014). Platelet-virus associated antibodies can also mediate platelet destruction (Zapata et al., 2014).

#### 4.2.1 Viral suppression of thrombopoiesis

Thrombopoiesis refers to the process of production of platelets. It primarily takes place in the bone marrow, where hematopoietic stem cells differentiate into polyploid megakaryocytes. Megakaryocytes are large cells that produce platelets. Production of platelets is regulated by the liver. The liver produces thrombopoietin (TPO). TPO stimulates the thrombopoietin receptors on megakaryocytes in the bone marrow (Gresele et al., 2017). This regulates the production and maturation. Viral infections lead to the suppression of thrombopoiesis, causing thrombocytopenia (Raadsen et al., 2021). Viruses can directly infect megakaryocytes, cells responsible for the production of platelets. This infection often results in megakaryocyte apoptosis, leading to decrease in the number of megakaryocytes available for producing platelets. Viral infection may also affect the maturation and the ploidy of megakaryocytes. Some viruses affect the hematopoietic stem cells, which are the precursors to megakaryocytes. This causes growth inhibition and apoptosis. Viruses can also indirectly affect platelet production by targeting the liver, which acts as the regulator for platelet production (Rasizadeh et al., 2026).

### 4.3 Coagulation pathway activation

#### 4.3.1 Fibrinolysis dysregulation

Fibrinolysis is a major process which is responsible for breaking down fibrin clots, this involves two main steps: Generation of plasmin with the help of plasminogen activators, other is subsequent digestion of fibrin by plasmin. Fibrin plays an active role in regulation by binding reactants and plasmin activity at the site of cleavage. Dysregulation in fibrinolysis leads to bleeding tendencies which can be categorized into primary and secondary hyperfibrinolysis (Wygrecka et al., 2022). Primary hyperfibrinolysis refers to the hemorrhagic disorders that are caused by direct quantitative or qualitative abnormalities of the fibrinolytic pathway involving proteins. Plasminogen Activator Inhibitor-1 (PAI-1) deficiency, α2-Plasmin Inhibitor (α2-PI) deficiency, Acute promyelocytic Leukaemia and Quebec Platelet disorder are some examples of primary hyperfibrinolysis (Table 3). When members of the fibrinolytic system have normal structure and availability but either act on fibrin that is more prone to lysis or their hyper-function is triggered in response to overt abnormal systemic blood clotting, this condition is referred to as secondary hyperfibrinolysis. Haemophilia, Factor XIII deficiency, Disseminated Intravascular Coagulation are the examples of secondary hyperfibrinolysis (Kolev and Longstaff, 2016).

**TABLE 3:** Molecular mechanisms underlying coagulopathy in viral haemorrhagic fevers.

Pathogenic mechanism	Viral factor involved	Host pathway affected	Coagulation outcome
Tissue factor induction	Viral glycoproteins	Extrinsic coagulation pathway	Hypercoagulation → DIC
Cytokine storm	TNF-α, IL-6, IL-1β	Endothelial activation	Vascular leakage, bleeding
Complement activation	Viral RNA/proteins	Complement– coagulation crosstalk	Thromboinflammation
NETs	Viral proteins	Intrinsic coagulation pathway	Immunothrombosis

### 4.4 Immune-mediated coagulopathy

Immune-mediated coagulopathy is a complex process in which the body’s immune response to a viral infection leads to blood clotting defects.

#### 4.4.1 Cytokine storm

Cytokine storm is an uncontrolled an excessive immune response which leads to inflammation and tissue damage. It also leads to Macrophage Activation Syndrome (MAS) which eventually causes damage to endothelium, and leads to thrombosis and microvascular permeability (Vadasz et al., 2020). During viral infection, proinflammatory chemokines attract and activate the neutrophils, which causes more tissue damage. The cytokine storm also involves the activation of helper T-cells (Th1 and Th2 cells) increasing the production of cytokines like IL-4 and IL-10. Accumulated macrophages receive stimulating signals through IFN receptors on their surface. This leads to production of more chemo-attractants, causing MAS (Vadasz et al., 2020).

#### 4.4.2 Complement activation

The complement system is an essential component of the innate immune system. It is involved in inflammatory responses, waste product removal, microbial opsonization, and direct pathogen destruction. Its activation is strictly regulated. Dysregulation of the Complement system can result in thrombotic microangiopathies (TMAs) and other disorders. The complement system primarily activates two pathways: Classical pathway and the Alternate pathway. The classical pathway starts when the C1 q component of the C1 complex attaches to antibodies bound by antigens, or to other targets such as surfaces of pathogens (Meri, 2013). Then the C1r and C1s activate the next components in the cascade: C2 and C4 (Meri, 2013). C3 convertase enzyme called C4bC2a is formed from the activation of C2 and C4 (Meri, 2013). It showcases a proteolytic activity towards C3. The alternative pathway generates another C3 convertase enzyme called C3bBb from activated C3 (C3b) and factor B (Bb) (Meri, 2013). The built-in amplification system is: activation of C3 promotes the production of new C3bBb convertases. The complement activation pathways are highly regulated. Controlling the activation of C3 is vital. The classical pathway C3 convertase (C4b2a) is regulated by the fluid phase inhibitor C4bp (Meri, 2013). The positive feedback activation of the alternate pathway requires a proper control mechanism. Factor H inhibits the activity of C3Bb and promotes C3b inactivation, both in fluid phase and on surfaces of pathogen (Vadasz et al., 2020; Meri, 2013).

#### 4.4.3 Neutrophil extracellular traps in immunothrombosis

NETs are important mediators of immunothrombosis. During viral infections, neutrophils are activated, and they release extracellular chromatin fibres. These interact with platelets, coagulation factors, and endothelial cells. NETs also increase the production of thrombin by activating factor XII and provide a structural framework for fibrin deposition. Furthermore, histones and proteases linked to NETs can directly harm endothelial cells and compromise vascular integrity. Excessive NET formation has been linked to both thrombotic problems and consumptive coagulopathy. In viral hemorrhagic fevers (VHFs), characterised by cytokine storms and endothelial activation, neutrophil extracellular traps (NETs) exacerbate the crosstalk between coagulation and inflammation, thereby increasing disease severity and causing organ dysfunction.

#### 4.4.4 Overall view of the molecular mechanism of coagulopathy

**FIGURE 2:**
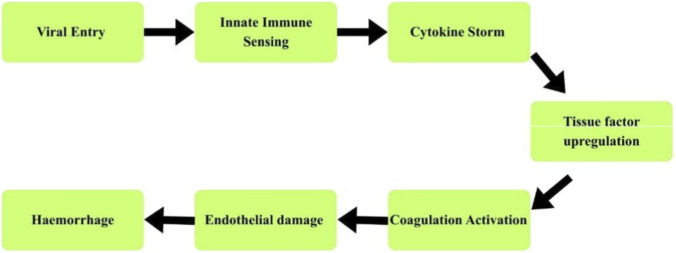
Molecular pathways leading to coagulopathy in viral hemorrhagic fevers.

The molecular mechanisms of coagulopathy in viral hemorrhagic fevers encompass a complex interplay of endothelial dysfunction, platelet activation, coagulation cascade dysregulation, and immune-mediated processes, including cytokine storm, complement activation, and neutrophil extracellular trap (NET) formation. These interrelated pathways collectively facilitate thrombin generation, fibrin deposition, and microvascular thrombosis, while concurrently predisposing individuals to consumptive coagulopathy and bleeding complications (Gatt et al., 2010). The convergence of these mechanisms underscores immunothrombosis as a pivotal aspect of VHF pathogenesis. A schematic view of these integrated molecular pathways is presented in Figure 2.

### 4.5 Role of viral proteins in VHFs

#### 4.5.1 Ebola glycoprotein (GP)

The EBOV GP gene produces many distinct proteins. These include the soluble glycoprotein (sGP), known as delta peptide or A-peptide. It also produces the small soluble glycoprotein (ssGP). These proteins influence the function of the endothelial cell barrier. The EBOV GP can impair the availability of specific integrins necessary for cell adhesion. This leads to liver damage and also affects blood clotting, causing endothelial dysfunction. sGP also has an anti-inflammatory function. It controls endothelial cell function and inflammatory response (Rivera et al., 2016).

#### 4.5.2 Dengue NS1

The Dengue virus non-structural protein 1 (NS1) is an important protein in the pathogenesis of dengue. It helps in viral replication, immune response control, and interactions with host factors (Perera et al., 2024). NS1 is involved in the replication of viral RNA expressed on the surface of the infected cells (Raj et al., 2025). Secreted NS1 (sNS1) levels in serum is a useful tool for diagnosing dengue infection. NS1 contributes to the pathogenesis of dengue by triggering the host immune response, since it is expressed on the surface of the infected cells (Azeredo et al., 2015). It also shows soluble complement-fixing activity. High levels of NS1 in the blood has been positively correlated with the development and severity of DHF(20,34). Secreted NS1 can bind to prothrombin and inhibit its activation to thrombin, causing prolonged activated Partial Thromboplastin Time (aPTT) and hemorrhage (Perera et al., 2024). NS1 antibodies can reduce the enzymatic activity of protein disulfide isomerase (PDI) and inhibit ADP-stimulated platelet accumulation (Perera et al., 2024). Cross-reactive antibodies against Dengue viral proteins like NS1, prM and E viral proteins can cause platelet dysfunction, macrophage activation, coagulation disorders and endothelial cell damage (Azeredo et al., 2015; Shen and Frenkel, 2007).

#### 4.5.3 CCHFV glycoproteins

Crimean-Congo Hemorrhagic Fever (CCHF) virus glycoproteins are significant in the pathogenicity of the virus during human infections. A highly variable mucin-like region is present at the N-terminal end of the glycoprotein. This region is riche in serine, threonine and proline, and undergoes O-glycosylation (Sanchez et al., 2002). This offers carbohydrate heterogeneity, which allows the virus to bind to various molecules (Doğan et al., 2018). The abundance of proline facilitates close packing of O-linked oligosaccharides, potentially offering protection from proteases. The CCHFV glycoproteins G1 and G2 are produced through the proteolytic processing of the M segment-encoded polyprotein. The N-terminal of mature G2 and G1 proteins are preceded by specific tetrapeptides, RRLL and RKPL, respectively. These tetrapeptides are highly conserved across CCHFV strains. CCHFV glycoproteins are critical for the virus’s interaction with host and cells. The mucin-like domain in their structure plays a vital role in the pathogenicity of the virus. The specific proteolytic processing of these glycoproteins by host cellular proteases is also important for their function (Doğan et al., 2018; Duru and Fışgın, 2026).

## 5 Biomarkers: sICAM, vWF, NS1 protein, cytokines

### 5.1 sICAM and VWF

Soluble Intercellular Adhesion Molecule-1 (sICAM) is a molecule that adheres to the cell, along with sE-selectin and sVCAM-1, is shed from endothelial cells after activation. These soluble forms are extensively researched as prognostic and diagnostic indicators for a range of infectious illnesses. It has often been observed that levels of sE-selectin, sICAM-1, and sVCAM-1 in patients at the time of sepsis diagnosis are higher than similar levels in non-septic. Some studies have shown that sICAM-1 can help in the predicting of mortality rate in septic patients with values showing impressive sensitivity and specificity. But some studies have shown contradicting results which have reported no association with mortality and varying predictive utility. Patients who were suffering from severe malaria had higher sICAM-1 levels compared to mild malaria and non-malarial illness. It has also been seen that it is a good predictor of outcome in the children with severe malaria (Page and Liles, 2013).

Von Willebrand Factor (vWF) plays an important role in maintaining blood flow and preventing coagulation (Page and Liles, 2013). It stabilizes platelet adhesion at vascular damage sites. When endothelial cells are activated, it is released from its storage in their Weibel-Palade bodies. Patients with critical sepsis the vWF levels were recorded higher than the normal people, these studies have shown link to mortality. Similar pattern is observed in patients who had severe malaria (Page and Liles, 2013; McElroy et al., 2014).

### 5.2 sNS1

During infection, NS1 is released in large amzounts via two primary oligomerization models that result in the creation of hexamers and tetramers, which differ depending on the flavivirus (Beltrami et al., 2025).

Increased permeability results from interactions between secreted soluble (s)NS1 oligomers and endothelial cells that compromise the integrity of the vascular barrier. Depending on the infecting virus, this interaction may affect host coagulation and fibrinolysis pathways, resulting in either procoagulant or anticoagulant consequences. NS1 interacts with interleukin-6 (IL-6) which helps in causing inflammation. sNS1 is a viral protein which has multiple functions, it influences both hemostatic and immune responses through different mechanisms with its effects based on specific flavivirus (Beltrami et al., 2025).

### 5.3 Cytokines

Cytokines are a broad, well-researched class of proteins that influence the immune response. They have been studied because of their various physiological and pathological processes. When the body is infected by VHFs, the elevated levels of cytokines are generally noticed. In severe viral infection, the amount of cytokine is more than the normal person (Chen et al., 2026), (Table 4).

**TABLE 4:** Laboratory biomarkers of coagulopathy in VHFs.

Biomarker	Change observed	Pathophysiological significance	Prognostic value	Limitations and variations
Platelet Count	Decreased	Platelet destruction, consumption, and bone marrow suppression	Strong predictor of severity and bleeding risk	May not correlate directly with bleeding due to rebalanced hemostasis; platelet function (not just count) is impaired; varies by virus (marked in dengue)
Prothrombin Time (PT)	Prolonged	Clotting factor depletion, liver dysfunction	Associated with poor outcomes and advanced disease	Can be influenced by liver injury rather than pure coagulopathy; may remain near normal in early compensated (SIC) phase
Activated Partial Thromboplastin Time (aPTT)	Prolonged	Intrinsic pathway impairment, factor consumption	Indicator of severe disease and DIC progression	Less sensitive in early disease; may be affected by anticoagulants or laboratory variability
D-dimer	Increased	Activation of fibrinolysis and clot breakdown	Strong predictor of mortality and disease severity	Elevated in both hyperfibrinolysis and thrombotic states; lacks specificity; high levels do not distinguish phenotype
Fibrinogen	Decreased (late stage)	Consumptive coagulopathy, fibrin formation	Predictor of bleeding risk in advanced disease	May be normal or elevated in early inflammation (acute phase reactant); decreases mainly in decompensated DIC or hyperfibrinolysis
Antithrombin (optional addition)	Decreased	Loss of endogenous anticoagulant activity	Associated with poor prognosis	Not routinely measured in all settings; influenced by liver function
PAI-1 (Plasminogen Activator Inhibitor-1) (optional)	Increased	Suppression of fibrinolysis (fibrinolytic shutdown)	Linked to organ failure and thrombosis	Not widely available; helps distinguish fibrinolytic phenotypes

**FIGURE 3:**
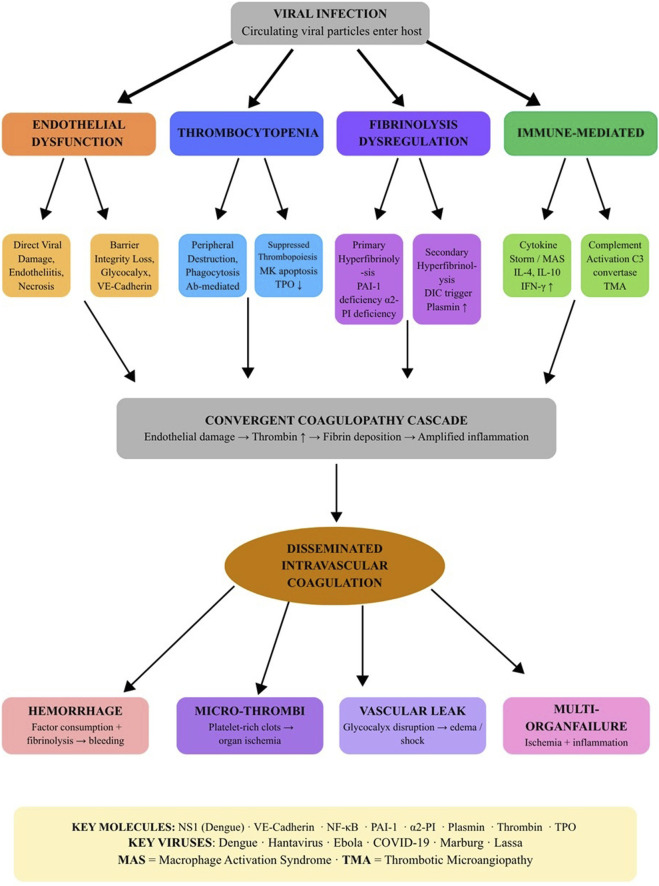
Molecular mechanisms of coagulopathy in viral hemorrhagic fevers (VHFs).

The development of coagulopathy in viral hemorrhagic fevers illustrates a complex and dynamic interaction among immune activation, endothelial dysfunction, platelet irregularities, dysregulated coagulation pathways, and the formation of neutrophil extracellular traps (NETs). These processes collectively contribute to the formation of thrombin, deposit fibrin, leak blood vessels, and eventually cause chronic coagulopathy. An integrated overview linking these molecular mechanisms of VHF-induced coagulopathy is presented in Figure 3.

## 6 Therapeutic interventions

### 6.1 Supportive management

#### 6.1.1 Fluid management

Supportive management majorly focuses on optimizing fluid therapy to address circulatory shock, a common and fatal pathophysiological pathway that is often observed in patients with VHFs. Typical clinical hallmark of viral hemorrhagic fevers is circulatory shock, which is brought on by significant intravascular volume depletion from gastrointestinal losses and extensive capillary leak. The cornerstone of treating these disorders is optimized fluid therapy. Restoring appropriate intravascular volume while preventing severe organ edema, which can result in respiratory failure, is the goal of fluid resuscitation. This balance is very important, in severe cases of dengue, fluid management is more conservative than in sepsis which prevents volume overload. Some innovative approaches to the treatment of VHF patients assist in maximizing fluid therapy while lowering the danger of virus transmission to medical staff and boosting the ability to provide sufficient supportive care. It is based on a fluid management algorithm that is managed remotely and directed by pulse pressure. Wireless blood pressure monitors, remote-controlled infusion systems, big resuscitation fluid bags, and a fluid resuscitation algorithm make up the intervention. For resuscitation the recommended fluid used is isotonic crystalloids like Ringer’s lactate (van Nieuw Amerongen and van Hinsbergh, 2002; Rizk et al., 2020).

#### 6.1.2 Platelet transfusion

Platelet transfusion in patients with Viral Hemorrhagic Fevers (VHF) should be restrictive and phenotypically driven because these diseases feature a balanced state of hemostasis with potential complications of both thrombosis and bleeding (Rizk et al., 2020). Transfusion therapy should only be used for hemorrhage or surgical interventions (typically <50,000/µL), whereas prophylactic transfusion of platelets solely according to the platelet level is not recommended in cases where immunothrombosis prevails, particularly in earlier or compensated stages of the disease. Platelet transfusions can be less effective in VHF due to dysfunctional platelets and their continuous consumption (van Nieuw Amerongen and van Hinsbergh, 2002; Kumar et al., 2015).

#### 6.1.3 FFP and cryoprecipitate

Frozen plasma (FP) is human donor plasma that is acquired via plasmapheresis or recovered from a single whole-blood donation, frozen within a certain amount of time following collection, and then stored at a certain temperature, usually −30 °C (Stanworth, 2007). Fresh frozen plasma (FFP) is plasma that has been frozen within 8 hours. Compared to FFP, FP may have somewhat lower levels of the labile coagulation factors V and VIII. Despite the fact that doctors often view both components (FFP and F24) as therapeutically similar (Stanworth, 2007).

The first concentrated form of anti-hemophiliac factor to be prepared practically was cryoprecipitate (Stanworth, 2007). Heavy proteins, such as FVIII, VWF, and fibrinogen, are prepared by carefully thawing FP at 1 °C–6 °C (Stanworth, 2007). The primary purpose of cryoprecipitate transfusion is as a concentrated source of fibrinogen. The plasma fibrinogen level is usually increased by up to 1 g/L (60–100 mg/dL) with an adult dose of about 10 single bags of cryo-precipitate made from units of whole blood (Stanworth, 2007). Because cryoprecipitate only contains considerable amounts of FVIII, VWF, fibronectin, FXIII, and fibrinogen, it should not be considered for transfusion as a more concentrated form of FP (for instance, when there are concerns about fluid over-load) (Stanworth, 2007). Although there is a lack of prospective trial data to determine the ideal usage of cryoprecipitate, it should only be used in individuals with proven isolated hypofibrinogenemia (Loftus, 2016; Stanworth, 2007; Srivalli and Lakshmi, 2012).

### 6.2 Antiviral therapies

Antiviral Therapies for VHFs are crucial for advanced levels of management. These therapies target the virus directly or control the host immune response to treat patients. Early treatment is often necessary for better patient outcomes. The field of antiviral therapies is progressing, with new drugs like Ribavirin and Flavipiravir available for use, with high efficacy. Novel drug discovery methods like HTS (High Throughput Screening) provide effective ways to find new compounds for the treatment of VHFs (Ippolito et al., 2012).

#### 6.2.1 Ribavirin

Ribavirin is one of the antiviral drugs used to treat VHFs. It is recommended for the prophylaxis and treatment of arenaviruses and bunyaviruses (Ippolito et al., 2012). It has been used to treat Lassa fever in patients for over 25 years (Ippolito et al., 2012). Studies have shown a significant decrease in the mortality among individuals treated with ribavirin. It is seen to be effective against Crimean-Congo Hemorrhagic Fever Virus (CCHFV). Ribavirin is one of the pharmacological options presents for the management and the treatment of VHFs. It helps in advanced life support and helps manage patients with multiorgan failure (Ippolito et al., 2012).

#### 6.2.2 Favipiravir

Favipiravir is a pyrazinecarboxamide compound, which is being studied as potential antiviral medications *in vivo* and *in vitro* for several types of VHFs. Promising results were seen in animal models against arenaviruses, yellow fever virus, Rift Valley Fever (RVF), West Nile virus, bunyaviruses, and yellow fever virus. Favipiravir shows a promising area of research in the development of new drugs for the treatment of VHFs, paving the way for more effective treatment strategies (Ippolito et al., 2012).

### 6.3 Targeted coagulation therapies

The concept of VHFs as thromboinflammatory disorders and hemostatic dysfunction is increasingly gaining recognition. The coagulation defect starts from the prothrombotic phase (sepsis-induced coagulopathy, SIC) and progresses to the consumptive phase (overt disseminated intravascular coagulation). These phases are usually associated with the development of endothelial dysfunction induced by cytokines and the formation of thrombi in the microvasculature. Therefore, anticoagulant strategies for VHF should target the modulation of the host response and not the virus itself (van Nieuw Amerongen and van Hinsbergh, 2002).

Anti-coagulation approaches that employ recombinant inhibitors of coagulation factors and endogenous anti-coagulants like activated protein C have shown encouraging, albeit nascent, success. As an illustration, recombinant nematode anti-coagulant protein c2 (rNAPc2), a tissue factor pathway inhibitor, and recombinant factor VIIa inhibitors have exhibited increased survival rates in Ebola-infected non-human primates (pre-clinical data), whereas activated protein C has been studied in severely infected states with multiple organ failure (Srivalli and Lakshmi, 2012; Ippolito et al., 2012). The therapeutic focus in this case is on tissue factor-mediated thrombin production and immunothrombosis, not the viral burden. Clinical application of such therapies is yet to be seen, as most information is based on pre-clinical studies or early-stage trials (Before clinical phase/Phase I-II), and questions exist about bleeding complications and subject selection criteria (Kumar et al., 2015).

Other emerging methods being studied involve the manipulation of cytokines or endothelium pathways, such as MIF inhibition or PAFR inhibition, but these are mainly based on experimental or observational findings. Anti-fibrinolysis, using tranexamic acid, has been tried in hemorrhagic complications due to dengue infection; however, its application is still debatable because it poses an increased risk of thrombosis in patients with fibrinolytic shutdown (McElroy et al., 2014). It should be mentioned that most of the targeted coagulation treatments have not received any strong evidence from randomized controlled trials (RCTs). Current information is too weak to recommend any of these interventions clinically. Hence, future research should focus on well-planned clinical trials and patient selection guided by biomarkers, so that these interventions do not cause further imbalance by promoting both thrombosis and bleeding (Stanworth, 2007).

### 6.4 Immunomodulators

#### 6.4.1 Corticosteroids

Corticosteroids are anti-inflammatory and antifibrotic agents. They have been used in suppressing inflammation of the lungs, especially in the advanced stages of the disease. They have also been used in antiviral therapy. They can downregulate the transcription of inflammatory cytokines, preventing a prolonged cytokine response. They also help in improving the dysregulated immune response seen in various VHFs. It can also increase the blood pressure in hypotensive patients (Low BP). There are some risks associated with the use of corticosteroids (Loftus, 2016; Ippolito et al., 2012). They can inhibit the immune response, potentially hindering pathogen clearance and promoting the replication of virus. Earlier studies on viral infection patients showed adverse effects of corticosteroids, including effects like avascular necrosis, diabetes, psychosis, delayed clearance of the virus and an overall increased risk in mortality. Unless there is additional indication (such as asthma, an exacerbation of COPD, or refractory septic shock), the World Health Organization (WHO) and the Centers for Disease Control and Prevention (CDC) do not typically suggest systemic corticosteroids for viral pneumonia (Raj et al., 2025). Although corticosteroids have strong anti-inflammatory effects that can lessen the severe inflammation associated with advanced VHFs, their use requires careful consideration of timing, dosage, and patient severity in order to get the potential benefits against risks such as viral replication and immune suppression (Rizk et al., 2020).

#### 6.4.2 Anti-TNF agents

TNF is crucial cytokine involved in nearly all acute inflammatory reactions, by inducing oxidative stress and inflammation. Macrophages, monocytes, B-cells are the main producers of TNF. TNF activation leads to the production of other inflammatory cytokines like IL-1 and IL-6. TNF levels are high during VHFs, due to the higher number of monocytes present during infection. Anti-TNF therapy includes using agents like Adalimumab and infliximab. They are commonly used in the management of rheumatoid arthritis, inflammatory bowel disease (Ippolito et al., 2012; Cron and Behrens, 2024). A single infusion of anti-TNF antibody can decrease the number of TNF in the blood, beneficial for VHFs. Some risks are involved with the use of anti-TNF agents. They may increase the risk of bacterial or fungal infections. Early initiation of anti-TNF is suggested to maximize effectiveness. Anti-TNF is considered a good option for managing the inflammatory stage of VHFs due to its wide availability, various dosage forms, and safety profile (van Nieuw Amerongen and van Hinsbergh, 2002).

#### 6.4.3 Complement inhibitors

One of the body’s two defenses against infections, the innate immune response, depends heavily on the complement system. Early after infection, innate immunity fights intruders. The complement system has a role in giving infections non-specific resistance. Immunoglobulin G (IgG) from human plasma is present in intravenous immunoglobulin (IVIG), which has been utilized to give immunization against viral illnesses. Inhibiting the activation and functioning of different innate immune cells and neutralizing activated complement components are two of the intricate ways that IVIG provides its therapeutic impact (van Nieuw Amerongen and van Hinsbergh, 2002). IVIG’s anti-inflammatory effects can also be attributed to its ability to bind to proinflammatory cytokines and antiviral antibodies. The effectiveness of IVIG in treating VHFs is still being assessed, despite its potential advantages in immune system modulation, particularly complement inhibition. Clinical improvement in severe VHF patients receiving high-dose IVIG has been documented in certain studies, including improvements in breathing rate and body temperature. The mortality rates of patients treated with IVIG and those who were not, however, did not differ significantly, according to other retrospective investigations. Since viral antibodies are not specifically included in current IVIG preparations, any advantages that are seen are probably the result of anti-inflammatory processes rather than direct antiviral efficacy. Although complement inhibitors, especially those administered via IVIG, have the potential to modulate the immune response during viral infections by blocking activated complement components, their direct antiviral efficacy and overall benefit in diseases are still being studied and are not generally advised (van Nieuw Amerongen and van Hinsbergh, 2002).

### 6.5 Emerging molecular therapies

#### 6.5.1 NS1 inhibitors

Glycosylation is a critical component for the function of NS1 and a validated antiviral target.

Inhibiting this N-linked glycan addition during the synthesis of endoplasmic reticulum can suppress NS1 secretion and downstream activity. Tunicamycin directly targets glycosylation and it impacts all the glycosylation sites including Asn207, which plays a critical role in maintaining the structure. Blocking glycosidase activity also affects the site of processing at Asn130 and Asn 175. Inhibitors like Cyanospermine (CST) and deoxynojirimycin (DNJ) reduce infectious virus (Watterson et al., 2016).

The minimal prerequisite for cleavage is an octapeptide that represents the last eight amino acids in NS1 (L/M-V-X-S-X-V-X-A) and is conserved throughout the flaviviral family. Indicates a shared target for broad-spectrum antiviral treatment (Watterson et al., 2016). Twelve cysteines in the NS1 protomer combine to produce six disulfide linkages that facilitate intra-domain stability. 12 Cysteines are conserved across the flaviviruses that infect birds and mammals, indicating the significance of these residues (Watterson et al., 2016). It is interesting to note that just ten of these cysteines are conserved for NS1 proteins that are exclusively infectious (Watterson et al., 2016). The resolved crystal structures for the West Nile virus (WNV) and dengue virus (DENV) NS1 have further elucidated the inter-residue connections, which were initially evaluated using mass spectrometry. Mutagenesis experiments have also shown how important these bonds are, suggesting that an antiviral approach that targets protein disulfide isomerase may be successful in blocking proper NS1 folding and function (Beltrami et al., 2025).

The molecular processes that underlie sNS1 action. Demonstrates that the pathogen-associated molecular pattern (PAMP) receptor TLR4 recognizes sNS1 using knockout cell lines and reconstituted *in vitro* receptor systems (Watterson et al., 2016). NS1-mediated cellular activation and endothelial leak can be prevented by targeting TLR4 with blocking antibodies or antagonists (Beltrami et al., 2025; Watterson et al., 2016).

#### 6.5.2 Therapies: host-directed

These therapies involve targeting the immune cells of the host rather than directly targeting the virus. It has been seen that Natural killer cells and macrophages are significant in determining the outcome of hemorrhage. The key differentiating between severe lethal infections and non-lethal, moderate diseases is differences in the timing and activation of NK cells. In order to lessen the severe consequences of VHFs, host-directed therapies seek to rebalance the host’s immune system, particularly by regulation of inflammatory cytokines like IL-6 and affecting the activation of critical immune cells like NK cells and macrophages (Ippolito et al., 2012).

#### 6.5.3 Endothelial barrier stabilizers

Endothelial barrier stabilizers are the substances that help in maintaining the barrier integrity of endothelial lining, which helps in preventing excessive vascular leakage. The identification of chemicals that stabilize barriers has made significant progress. Sphingosine 1-phosphate (S1P), adreno-medullin (ADM), Ang1, and the second messenger cyclic AMP (cAMP) are currently recognized endogenous barrier-improving substances. Recent studies have identified several endogenous compounds that contain compounds which has proven barrier improving properties, which offers promising future therapies.

Some of the key Endogenous barrier stabilizers are Cyclic (AMP), Adrenomedullin (ADM), Sphingosine 1-phosphate, Hepatocyte Growth Factor (HGF), Activated Factor XIII and Angiopoietin and Hypoxia Inducible Factor 1 (HIF1). These endogenous compounds and pathways offer impressive and promising new targets for the pharmacological interventions that help in the stabilization of endothelial barriers and function and counteract the vascular leakage in different kinds of disease (Doğan et al., 2018; van Nieuw Amerongen and van Hinsbergh, 2002).

**FIGURE 4:**
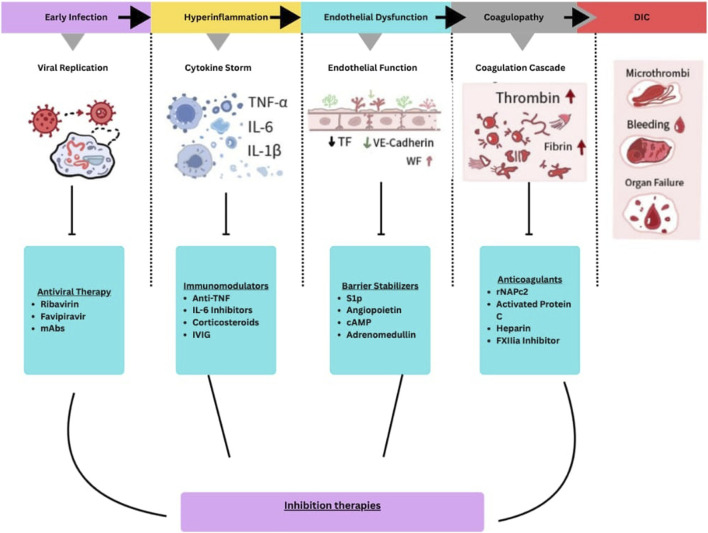
Therapeutic targets in virus-induced coagulopathy.

This integrated perspective reflects the central role of immune–coagulation crosstalk in disease progression. A comprehensive overview of these therapeutic targets and their corresponding intervention strategies is illustrated in Figure 4 and Table 5.

**TABLE 5:** Targeted therapeutic interventions for virus-induced coagulopathy.

Therapeutic strategy	Target pathway	Example agents	Current status
Anticoagulants	Tissue factor pathway	rNAPc2, heparin	Experimental/limited use
Antiviral therapy	Viral replication	Ribavirin, monoclonal antibodies	Approved/clinical use
Immunomodulators	Cytokine signaling	Tocilizumab, corticosteroids	Investigational
Endothelial stabilizers	Vascular integrity	Statins, angiopoietin modulators	Emerging
Supportive transfusion	Platelet/coagulation factors	Platelets, FFP, cryoprecipitate	Standard care

## 7 Future directions and research gaps

Precision immunomodulatory therapeutic research is increasingly trending within VHF treatment. Current research tends to adopt a precise strategy based on thromboinflammation, immunothrombosis, and unregulated hemostatic processes (Stanworth, 2007). Despite efforts made to enhance patient outcomes, several shortcomings have been highlighted regarding translation from theory to practical applications.

One important aspect that needs improvement in current research literature involves the use of old definitions of Disseminated Intravascular Coagulation (DIC), which describes it as a consumptive and bleeding problem. Recent models have defined that infection-related coagulopathy is a condition that ranges from Sepsis-induced coagulopathy (SIC) to DIC. This shows that it is a dynamic process that is continuously changing (Srivalli and Lakshmi, 2012; Ippolito et al., 2012; Cron and Behrens, 2024).

In this context, VHFs are classified as conditions with coagulation disorders, which include:

- Non-overt DIC (compensated DIC): Early stage disease, characterized by enhanced thrombin production, relatively normal or slightly impaired platelet counts, and prothrombotic status driven mainly by immunothrombosis and fibrin formation in microvasculature.
- Overt DIC (decompensated DIC): Late-stage disease, characterized by utilization of platelets and coagulation factors, causing symptoms of both thrombosis and hemorrhage (Watterson et al., 2016).

Also, contemporary studies have revealed the significance of unique fibrinolytic profiles, which are generally neglected in VHF research:

- Fibrinolysis-suppression profile (“fibrinolytic shutdown”): Characterized by high PAI-1 levels, creating persistent microthrombi and organ dysfunction.
- Fibrinolysis-hyperactivation profile: Marked by excessive plasmin formation, causing major hemorrhagic events, especially during later stages of VHF such as dengue (McElroy et al., 2014).

It is important to identify these phenotypes, since the approach for treatment can vary widely according to the dominant hemostatic dysregulation. Antifibrinolytics like tranexamic acid can be effective for hyperfibrinolysis, while they can do harm when there is a shut-down of fibrinolysis (Duru and Fışgın, 2026; Dorgalaleh et al., 2021; Zaidi and Rout, 2025; Toulon et al., 2021).

Another important scientific knowledge gap involves understanding how best to time and personalize immunomodulatory therapy. The efficacy of therapies like cytokine inhibitors, anticoagulant medications, or antivirals is heavily influenced by the phase of the disease. Early-stage interventions can focus on stopping viral replication and activating innate immunity, while later interventions can focus on modulating coagulation systems, endothelial damage, and inflammatory processes (Ippolito et al., 2012; Cron and Behrens, 2024).

Limited knowledge on pathogenesis is an impediment towards developing adequate treatments for Viral Hemorrhagic Fever (VHF). While cytokine storms and viral immune evasion have been noted as contributing factors in pathogenesis, there is limited knowledge on the interaction of both elements and how these interact with coagulation processes (Kumar et al., 2025). Specifically, more needs to be known on the effects of monocyte tissue factor production, thrombin generation, and endothelial cell activation through PARs. Despite all these problems, there are certain therapies that can be explored as potential solutions. First, inhibition of coagulation pathways through the use of recombinant factor VIIa and activated protein C in experimental animal studies involving Ebola virus showed improved survival rates. This indicates an important role of coagulation pathways in thromboinflammation and not just clot prevention. On the other hand, cytokine therapy, such as MIF inhibitors, might offer another avenue for treatment (Zaidi and Rout, 2025; Toulon et al., 2021; Weitz et al., 2017; Chen et al., 2026; McElroy et al., 2014).

Passive immunotherapy, especially the convalescent plasma treatment, has proven to be variably effective with restrictions in terms of timing, necessary neutralizing antibody levels, and practical issues. Gene silencing methods like siRNA therapies have been shown to provide promising results in pre-clinical Ebola studies; however, further applications in the clinical setting remain impractical due to financial and manufacturing issues (Srivalli and Lakshmi, 2012; Ippolito et al., 2012). Furthermore, small-molecule library screening presents an approach worth considering with regard to the discovery of new drugs for modulation of immune system and anticoagulation. Future treatments will become more oriented toward restoration of overall hemostatic homeostasis rather than individual components of the blood coagulation process. In order to improve the quality of current clinical procedures, further research needs to concentrate on implementing standardized methodologies developed by organizations like the International Society on Thrombosis and Haemostasis, e.g., DIC and Sepsis-Induced Coagulopathy (SIC) scoring systems (Cron and Behrens, 2024; Watterson et al., 2016; Kumar et al., 2025; Woodson et al., 2013).

Moreover, the classification of patients according to coagulation and fibrinolytic phenotypes, the creation of standard and adapted treatment strategies for resource-poor regions, and the search for drug combinations aimed at viral replication, immune dysregulation, and coagulation cascades need to be considered (Woodson et al., 2013). In conclusion, the improvement of VHFs’ management is impossible without moving from the conventional approach to coagulopathy towards the systemic concept of thromboinflammation and hemostasis imbalance.

## 8 Conclusion

This paper is about coagulopathy and mentions its role in viral hemorrhagic fevers (Ebola, Dengue, Marburg and CCHF). It gives an overview about how viruses interact with the body and causes bleeding disorders which can ultimately cause death in severe stages of disease. This paper also includes a brief explanation of molecular mechanisms and therapies which covers how endothelial damage, Cytokine storm and DIC are caused due to the infection of VHFs.

Inflammation-coagulation crosstalk explains how cytokines like TNF-alpha, IL-6 activate the endothelium layer which causes vascular leak and are also responsible for TF upregulation (Falanga et al., 2008). In VHFs virus host cells infects the macrophages and triggers the cytokine storm which results in the breakdown of endothelial barrier, and glycocalyx shedding which is observed in dengue NS1, platelet Dysfunction and thrombin bursts leads to fibrin deposition which causes more bleeding. The viral NS Protein and GP protein of ebola contribute majorly in coagulopathy along with it they cause organ damage like lymphoid depletion. Cytokine storm and complement activation is responsible for causing the immunothrombosis. Together they form DIC, in this the loss of clotting factors is observed which leads to hemorrhage. Diagnostic markers like PT/APTT, high D-dimer, low platelets and fibrinogen levels help us in predicting the severity of VHF.

Fluid management (crystalloids like Ringer’s lactate), platelet/FFP transfusions are important for supportive care and therapy. But there are some risks associated with prophylactic platelets which has impact on mortality rates in some cases. Immunomodulators like anti-TNF, corticosteroids and component inhibitors can control the cytokine storm and similarly this also has risk like it can suppress the immunity causing immunosuppression. Emerging therapies like NS1 inhibitors (glycosylation blockers), host directed therapies and endothelial stabilizers (S1P, ADM) can be game changer in the future. There are still a lot of gaps in this field, exact timing of therapeutics patient-specific protocols and novel drug clinical trials still has to be addressed. Personalized medicines based on biomarkers like sICAM, vWF, cytokines are important for further understanding of targeted therapies which can mitigate both bleeding and thrombosis.
